# Molecular and Histopathological Changes Associated with Keratoconus

**DOI:** 10.1155/2017/7803029

**Published:** 2017-01-30

**Authors:** Mariam Lotfy Khaled, Inas Helwa, Michelle Drewry, Mutsa Seremwe, Amy Estes, Yutao Liu

**Affiliations:** ^1^Department of Cellular Biology and Anatomy, Augusta University, Augusta, GA, USA; ^2^Department of Ophthalmology, Augusta University, Augusta, GA, USA; ^3^James & Jean Culver Vision Discovery Institute, Augusta University, Augusta, GA, USA; ^4^Center for Biotechnology and Genomic Medicine, Augusta University, Augusta, GA, USA

## Abstract

Keratoconus (KC) is a corneal thinning disorder that leads to loss of visual acuity through ectasia, opacity, and irregular astigmatism. It is one of the leading indicators for corneal transplantation in the Western countries. KC usually starts at puberty and progresses until the third or fourth decade; however its progression differs among patients. In the keratoconic cornea, all layers except the endothelium have been shown to have histopathological structural changes. Despite numerous studies in the last several decades, the mechanisms of KC development and progression remain unclear. Both genetic and environmental factors may contribute to the pathogenesis of KC. Many previous articles have reviewed the genetic aspects of KC, but in this review we summarize the histopathological features of different layers of cornea and discuss the differentially expressed proteins in the KC-affected cornea. This summary will help emphasize the major molecular defects in KC and identify additional research areas related to KC, potentially opening up possibilities for novel methods of KC prevention and therapeutic intervention.

## 1. Introduction

**Figure 1:**
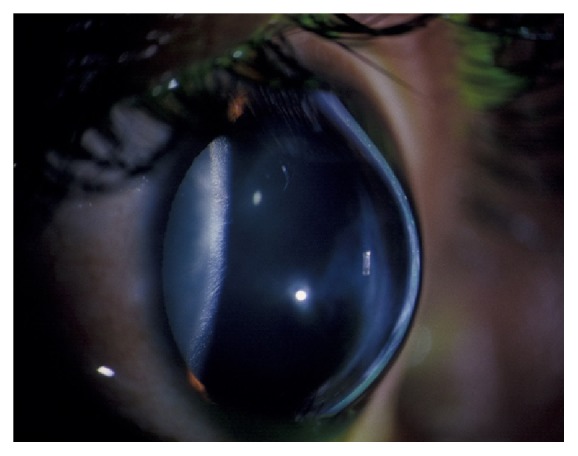
Cone shaped phenotype of the cornea in a keratoconus patient.

The cornea is the outermost avascular and transparent part of the eye consisting of epithelium, Bowman's layer, stroma, Descemet's membrane, and endothelium. In 2013, a novel collagenous, acellular layer, the Dua layer, was identified between corneal stroma and Descemet's membrane [1]. Each layer has a specific important function, and a defect in any of these layers can lead to corneal disorders. The most common corneal ectatic disorder and a leading indicator for corneal transplantation in developed countries is keratoconus (KC) [2, 3]. KC is a bilateral, progressive ectatic disease where the cornea becomes cone shaped due to significant thinning of the corneal stroma (Figure 1). Visual impairment develops from myopia and irregular astigmatism [4]. Early forms of KC can be more accurately detected and potentially quantified in a reproducible manner with corneal topography [5].

Although KC has long been described as a noninflammatory disorder, recent reports have indicated possible inflammatory mechanisms [6–8]. KC usually starts at puberty and progresses until it stabilizes in the third or fourth decade. An inverse correlation has been noticed between age and KC severity [9, 10]. The earlier the onset of KC, the more severe the clinical phenotypes. KC appears in all ethnicities and has no gender preference [5, 11]. The prevalence of KC varies greatly worldwide; it was reported at 0.0003% in Russia [12], 0.086% in Denmark [13], 0.249% in Iran [14], and 2.3% in central India [15]. Not only do geographical variations change KC prevalence, but also the source of the collected data does. For example, in the USA, the prevalence of KC was found to be 600/100,000 in a population based study [16] and 54.5/100,000 in a hospital records based study [17].

Many reviews have previously summarized the genetic studies linked to KC incidence [18, 19], whereas others have discussed the various advances in treatment modalities [20, 21]. Despite recent advances in KC research, the molecular and pathological mechanisms of KC still remain unclear. To our knowledge, there is currently no comprehensive article that collectively summarizes the clinical and histopathological phenotypes associated with molecular and biochemical changes in KC. We hope that a better understanding of pathological changes associated with KC may promote further research to identify novel therapeutic targets that could stop or delay KC progression.

### 1.1. Clinical Signs of KC

**Figure 2:**
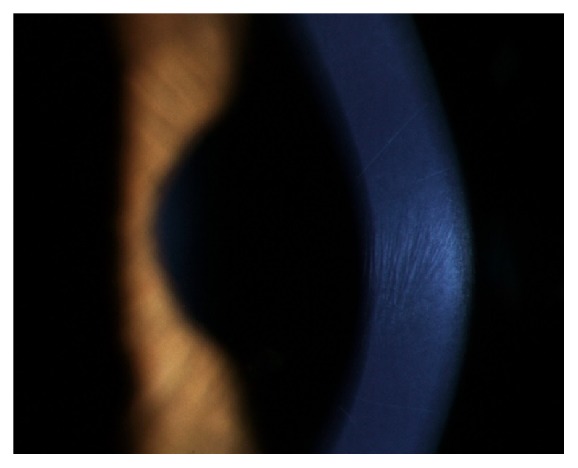
Sign of Vogt's striae showing fine vertical lines in deep stroma and Descemet's membrane of a keratoconus patient.

**Figure 3:**
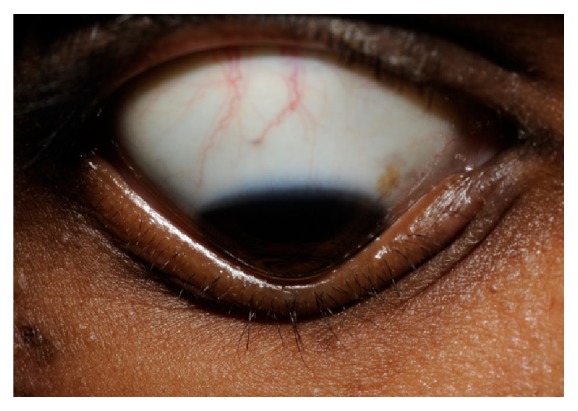
Münson's sign in a keratoconus patient which appears as bulging of the lower lid during downgaze.

Clinically, the primary symptoms of KC are reduced visual acuity, photophobia, monocular diplopia, and glare. Due to disease progression, KC patients usually need frequent adjustment of their spectacle correction, and often vision cannot be corrected to 20/20 with spectacles alone [22]. In moderate to advanced KC, slit lamp examination can often capture clinical signs of KC, such as Fleischer's ring (iron lines partially or completely surrounding the cone), Vogt's striae (fine vertical lines in deep stroma and Descemet's membrane, Figure 2), corneal thinning, and Münson's sign (bulging of the lower lid during downgaze, Figure 3). Other accompanying signs that might appear are increased visibility of corneal nerves, apical thinning, anterior stromal clearing lines, subepithelial fibrillary lines, and central or eccentric corneal protrusion [5]. Corneal topography, a key diagnostic method for KC, has greatly aided in diagnosis and treatment of KC and forme fruste, the subclinical presentation of KC, leading to earlier treatment of these patients.

### 1.2. Etiology

KC is a complex multifactorial disorder, and changes in numerous genes and environmental factors are thought to be responsible for the disease development and progression [23].

#### 1.2.1. Genetic Factors

The majority of KC cases are sporadic; however, 6–23.5% of keratoconic patients have a positive family history [24]. First-degree relatives of KC patients have a risk of developing KC that is 15–67 times higher than the general population [25]. The suggested pattern of inheritance in these familial cases is mostly autosomal dominant (reviewed in [18]). Monozygotic twins have been reported to be concordantly affected with KC, rather than discordant for KC, which is considered important evidence for genetic contribution in the pathogenesis of KC [26]. Wang et al. have suggested that KC is inherited likely due to a major gene defect [27], while Kriszt et al. have indicated that KC is a complex non-Mendelian disease [28]. Family-based linkage analyses have identified at least 17 genomic loci from 12 different studies [29–40]. Mutations in the* MIR184 *gene have been found to cause KC, but the majority of the mutations remain to be identified (reviewed in [18]).

#### 1.2.2. Environmental Factors

Besides genetic factors, many environmental factors have been documented as contributors to KC pathogenesis in patients with and without any family history [18]. These factors include contact lens wear [20], vigorous eye rubbing [41, 42], atopy [43–45], ultraviolet light exposure, and other factors that can be related to increased oxidative stress in the cornea [46, 47].

### 1.3. Other Disorders Associated with KC

Usually KC is thought to be a sporadic disease; however, it has been described in association with many syndromes and diseases. Down syndrome patients were found in several studies to develop KC at a higher frequency [11, 25, 48–52], while other studies reported the absence of KC in this subset of patients [53–55]. Interestingly, studies of Down syndrome patients who are less than 18 years old observed less or no incidence of KC compared to controls [52–55], while adult Down syndrome patients had a greater prevalence of KC [50, 51, 56, 57]. KC has also been associated with Leber congenital amaurosis. Up to 30% of Leber congenital amaurosis patients were reported to have KC, possibly due to the mechanical effects produced by eye rubbing [58–61]. Previous studies have also linked KC with many connective tissue diseases, including but not limited to Ehlers–Danlos syndrome [62], osteogenesis imperfecta [63], mitral valve prolapse [64, 65], Mediterranean fever [66], and joint hypermobility disease [67]. In contrast, another study has shown lack of association between KC and mitral valve prolapse or joint hypermobility [68]. A negative association has been identified between diabetes mellitus and KC (i.e., a reduced risk of developing KC in diabetic patients) [11, 69, 70]. In patients with diabetes mellitus, high levels of glucose may cause glycosylation of corneal fibers and induce collagen cross-linking in the stroma, in turn preventing biomechanical weakening of the cornea and reducing the risks of ectasia and KC [69, 71]. Additionally, Nemet et al. have reported a strong correlation between KC and allergic immune disorders, as well as autoimmune diseases [43], adding evidence to the positive relationship between KC and inflammation. Dry eye symptoms, such as lowering in tear secretion, tear film break up time, and corneal sensitivity, have been reported in KC patients [72–74]. Impairment of the corneal sensory nerve activity [74], decreased mucin production in tears [73], or elevated inflammatory mediators have been proposed to account for these dry eye associated symptoms in KC patients [72].

## 2. Histopathological Abnormalities in KC

The cornea is composed of six distinct layers: the outer stratified, squamous nonkeratinized epithelium, the acellular Bowman's layer, the inner connective tissue stroma with its resident keratocytes, the pre-Descemet's Dua layer, Descemet's membrane, and the cuboidal monolayered endothelium. The cornea is surrounded anteriorly by the tear film and posteriorly by aqueous humor. Maintenance of corneal shape and transparency is critical for optimizing the eye's refractive power [75]. Researchers have used a variety of different advanced techniques to evaluate major morphological corneal changes in KC patients [76–82]. Light microscopy, confocal microscopy, and optical coherence tomography (OCT) have been used to examine the cornea in vivo [76, 83–86], while electron and light microscopy have been used to investigate fixed and processed corneal tissues in vitro [84, 87, 88].

An in vitro study with 95 KC-affected cornea specimens has categorized keratoconic corneal tissues into two microscopic patterns: typical and atypical [88]. The typical pattern has both stromal and central epithelial thinning with multiple Bowman's layer breaks, while the atypical one lacks breaks in Bowman's layer and has less thinning of the central epithelium [88]. The typical pattern has been identified in more than 80% of the corneas and is present in 72% of the patients with bilateral corneal transplants. Using OCT, an in vivo study by Sandali et al. proposed a classification system for KC using five distinct stages to characterize the keratoconic progression [85]. Patients in stage 1 have a thinner corneal epithelium and stroma at the conus than control. In stage 2, hyperreflective anomalies in Bowman's layer are noticed with thickening epithelium and opaque stroma. In stage 3 there is increased epithelial thickening and stromal thinning with disruptions in Bowman's layer. Stage 4 shows pan-stromal scarring, and finally, stage 5 is considered as the acute form of keratoconus (hydrops) with Descemet's membrane rupture and total corneal scar [85]. Brautaset et al. have proposed that KC is a pan-corneal thinning disorder based on the corneal thinning appearance in the peripheral and central ectatic region [86].

### 2.1. Corneal Epithelium

Corneal epithelium functions as a diffusion barrier to water and solutes and as a mechanical barrier to microorganisms. It is one of the most highly innervated parts of human body and accounts for approximately 10% of the corneal thickness. The epithelial cells tightly adhere to each other and to the basement membrane [75]. Various studies have reported that basal epithelial cells in KC patients exhibited enlargement, irregular arrangement, and a significant reduction in cell density when compared to the control group [77–79, 88]. Though epithelial thickness is thought to be negatively correlated with the KC severity [77, 89], other studies have demonstrated either no significant change of corneal epithelium [80] or thickened corneal epithelium in KC patients [76, 90, 91]. Corneal epithelial apoptosis, resulting in epithelium thinning, could result from chronic epithelial injury due to various environmental risk factors and could result in the release of apoptotic cytokines [87, 92].

### 2.2. Nerve Fibers

Increased visibility of the nerve fibers on slit lamp examination is one of the characteristic signs of KC [93, 94]. Although thinning of the cornea is the main reason for this increased nerve visibility [95], several subsequent studies have identified morphological abnormalities in corneal nerves. In KC, the architecture of the subbasal corneal nerve plexus (located between the Bowman layer and the basal epithelium) has been shown to have a fragmented plexus [96] and a reduced central nerve fiber density [97]. Additionally, localized nerve thickening has been observed in close proximity to breaks in Bowman's membrane with wrapping of anterior keratocytes around the nerve [98, 99].

### 2.3. Bowman's Layer

Bowman's layer, also known as the Anterior Limiting Lamina, is an acellular collagen fibril matrix at the interface between the corneal epithelium and the stroma [100]. The actual function of Bowman's layer is still unknown. Many mammals have no Bowman's layer, yet corneal stability is not compromised. In KC, cellular components have been observed in Bowman's layer, despite typically being acellular [101]. Other studies have demonstrated ruptures within Bowman's layer [81, 88] and the coexistence of a proliferative collagenous tissue derived from the anterior stroma just beneath Bowman's layer [102]. Isolated Bowman's layer transplantation has reduced and stabilized corneal ectasia in eyes with progressive and advanced KC [103], but it remains unknown whether Bowman's layer contributes to the pathogenesis of KC.

### 2.4. Stroma

Stroma (Substantia Propria) accounts for approximately 80% of the cornea thickness [100]. It is a highly organized collagenous matrix consisting of multiple collagenous lamellae and keratocytes. Keratocytes are specialized mesenchymal cells that reside between lamellae. Within each lamella, collagen fibrils are parallel, tightly packed, and highly uniform in diameter. This organized architecture is responsible for the transparency of the cornea, and any disruption in this organization results in a severely opaque cornea [104]. Stromal keratocytes secrete and maintain the stromal matrix components and account for 10% of the stromal volume [75]. In KC, a significant decrease in the number of lamellae without thickness alteration [105, 106] and the appearance of nonkeratocyte cells and tissue debris have been demonstrated [87]. These nonkeratocytes are agranular and may have a role in the break down and phagocytosis of corneal tissues [87]. Significant reduction has been reported in anterior and posterior keratocyte density [78, 80, 81, 87, 99].

The collagen lamellae at the anterior stroma of a normal cornea are interwoven and narrow and form a steep angle with Bowman's layer. With progression towards Descemet's membrane, these lamellae become wider and their angle relative to Bowman's layer becomes flattened [107]. However, in KC collagen lamellae are wider and form a smaller angle with Bowman's layer. It has been suggested that collagen lamellae are expanded in association with protrusion of the cone [107]. Stromal lamellae in KC-affected cornea undergo splitting into multiple bundles of collagen fibrils with loss of the anterior lamellae [108]. Since the ACTB gene encoding *β*-actin has been shown to be downregulated in KC [109, 110], these data suggest that the decrease of stromal keratocytes in KC may contribute to the reduced expression of *β*-actin, destabilization of cytoskeleton, and finally the thinning and weakening of the stroma [110].

### 2.5. Descemet's Membrane

Descemet's membrane, also referred to as the Posterior Limiting Lamina [100], is the membrane which separates the endothelial layer from the stroma. Like Bowman's layer, it is an acellular layer and is not continuous with the collagen fibrils of the stroma. Ruptures of Descemet's membrane have been observed in KC [111]. The common morphological folds and irregularities in Descemet's membrane do not show any consistent alterations with its extracellular matrix components [47]. Apparent Descemet's folds have been found in 8.3% of the KC cases associated with pleomorphism (variation in shape) or polymegethism (variation in size) of endothelial cells [91]. Rupture in Descemet's membrane with entering of aqueous humor into corneal epithelium and stroma is a serious complication for KC, which is known as acute corneal hydrops [112]. Sutures in either Descemet's membrane or Dua layer have been reported as an efficient surgical treatment for acute hydrops [113, 114].

### 2.6. Endothelium

The endothelium is a monolayer of regularly sized polygonal cells, which mainly function to regulate the water content of corneal stroma. In several studies, this layer does not exhibit any changes during KC progression [79, 83, 85, 115]. Conversely, some studies have reported slight increase in endothelial cell density in KC [72], while others have shown significant decrease in moderate to severe KC [78, 91]. Several studies have demonstrated that endothelial cells in the peripheral region have a higher density than those in the central region [80, 116–119], suggesting that human corneal endothelial stem/progenitor cells are mainly distributed in the periphery [120]. These studies emphasize the necessity of determining whether the endothelial morphological changes in KC are within the center or the periphery of the cornea [76, 83].

Additionally, clinical treatment supports the lack of endothelium involvement in KC pathogenesis. Penetrating keratoplasty (PK) is a surgical procedure involving the removal of a full thickness portion of corneal tissue. In patients with KC, deep anterior lamellar keratoplasty (DALK) is considered an excellent alternative surgical option to PK. DALK preserves patient's endothelial layer, reducing their risk of graft rejection [121]. The preference of DALK in KC patients suggests that the most affected corneal layers in KC are the corneal epithelium and stroma. Histopathological changes in corneal layers other than epithelium and stroma may be secondary to the epithelial and stromal wound healing process [47, 88, 122].

Collagen cross-linking (CXL) and intrastromal corneal ring segments implantation (ICRS) are alternative procedures for KC treatment. CXL uses riboflavin and ultraviolet-A rays to stop the progression of KC via increasing corneal biomechanical resistance [123, 124]. Postoperatively, confocal microscopy has been used to examine corneal changes that may occur after CXL. Histopathological changes such as demarcation lines, keratocytes apoptosis, and stromal edema have been reported to disappear within 6 months, with improvement in the visual acuity; thus CXL has been considered as an effective and safe procedure [125, 126]. ICRS implantation is a reversible, minimally invasive procedure for moderate KC without central corneal opacities. ICRS corrects corneal ectasia via shortening the cone length, causing corneal flattening to the periphery [127]. Improvements in the refractive power, topographic measurements, and optical quality have been reported postoperatively, with an increase in contact lens tolerability [127–129]. Complications, such as segment extrusion, segment migration, or shadow effects, have been found to be rare among patients [127].

## 3. Disrupted Corneal Homeostasis and KC

KC is known to be degenerative and progressive. Homeostasis of the corneal microenvironment is controlled and balanced through various molecular mechanisms; however, the main molecular mechanisms that contribute to the structural and biochemical abnormalities in KC are still unclear. In this section, we summarize the documented molecular changes associated with KC and their impact on the integrity and transparency of the cornea.

### 3.1. WNT and HH Proteins

The WNT and Hedgehog (HH) proteins are secreted proteins that regulate a variety of developmental processes in vertebrates and invertebrates by inducing transcriptional or morphological changes in responding cells [130, 131]. It is well documented that WNT and HH signaling controls stem cell differentiation [132–134]. Inappropriate activation of WNT and HH signaling pathways can lead to various diseases [135, 136]. Few studies have been done to determine the role of those signaling pathways in the progression of the KC [137, 138].

Cornea epithelial cells undergo continuous renewal from limbal stem cells [139], and a deficiency in self-renewal can lead to deleterious effects on corneal wound healing and surface integrity [140]. Furthermore, improper differentiation of these stem cells can give rise to keratinized, nontransparent corneal epithelium [137]. An in vitro study has shown that the HH and WNT pathways are necessary to maintain corneal endothelial cell integrity and structure [120]. Knock-down of WNT7A switches corneal epithelial cells to skin-like epidermal cells and negatively affects the transparency of the cornea, suggesting its involvement in corneal epithelium differentiation [137]. Recently, a missense coding variant (rs121908120, c.1145T>A, p.228Phe>Ile) in the* WNT10A* gene has been associated with KC via decreased corneal thickness [138]. An intronic variant rs10453441 in the* WNT7B* gene has been associated with central cornea thickness [141]. These data illustrate the functional involvement of WNT pathway components in the pathogenesis of KC.

### 3.2. Cellular Adhesion Molecules (CAM)

Cellular adhesion molecules (CAM) are cell surface receptors that play important roles in various cell-cell and cell-extracellular matrix interactions in the cornea. KC is associated with various defects in corneal layer structure and integrity, which may be related to a disturbance in the expression of CAMs in the cornea. It has been previously reported that CD34, a CAM and a hematopoietic stem cell marker, is expressed in normal human corneal keratocytes [142]. By studying healthy versus diseased corneal samples including KC, it has been demonstrated that the loss of CD34 immunoreactivity seems to be a constant feature and early event in KC [143].

Another CAM, Desmoglein 3 (DSG3), is a desmosomal cadherin that mediates cell-cell adhesion via desmosomes [144]. Nielsen et al. have reported significantly increased DSG3 in the mRNA and protein levels in all of the KC samples studied [145].

Laminin and fibronectin are CAMs essential for the binding of basal epithelial cells to the basement membrane via integrins. Earlier studies have demonstrated overexpression of fibronectin in scarred areas of the anterior KC cornea [47, 146]. Similarly, Deng and colleagues have observed increased staining of fibronectin in the basement membrane of corneal epithelial cells, especially in regions of scarring [147]. Upregulation of DSG3, laminin, fibronectin, and other types of CAM in KC could be downstream events of the wound healing cascade.

Corneal wound healing is a complex process. It involves the integrated actions of multiple growth factors, cytokines and proteases produced by epithelial cells, stromal keratocytes, and inflammatory cells [148]. Following an epithelial insult, various cytokines are released to induce a cascade of events in an attempt to repair the epithelial defect and modulate remodeling of the stroma, minimizing loss of transparency and function. However, an unregulated process of wound repair could result in disease [148]. In 2001, Deng et al. have proposed that a process similar to wound healing may contribute to the changes seen in the KC patients [147].

### 3.3. Collagen and Proteoglycans in KC

The cornea has at least 11 types of collagen. Within the corneal extracellular matrix (ECM), collagen interacts mainly with two types of proteoglycans: keratan sulfate (the major proteoglycan in the cornea by 60%) and chondroitin/dermatan sulfate. Proteoglycans consist of a core protein and a glycosaminoglycan side chain. In keratan sulfate, the main core proteins are keratocan, which is unique to the cornea, lumican, and fibromodulin. For the chondroitin/dermatan sulfate, the primary core proteins are decorin and biglycan [75, 149, 150]. As mentioned earlier, the transparency of cornea requires uniform orientation of collagen fibers in the corneal matrix, and normal expression of proteoglycans is essential for this organized architecture [75].

In KC, components of the ECM have been shown to have altered expression levels or abnormal localizations [47, 151]. Many proteomic studies have identified differences in the abundance of proteins between normal and keratoconic corneas (Table 1). Lumican and keratocan have been shown to be significantly decreased in KC [152, 153], yet keratocan has been reported to be highly expressed in the stroma of KC compared to normal or other diseased cornea samples, such as from Fuchs' corneal dystrophy or pseudophakic bullous keratopathy patients [154]. Joseph and colleagues, using the shotgun proteomics method, have further confirmed a 2.4-fold increase of the stromal keratocan in KC patients [155]. Collagen types I, III, V, and XII have been identified to have a lower expression level in KC [152]. Reduction in the collagen components might be related to a defect in the hydroxylation of proline due to endoplasmic reticulum stress or chaperone defects [152]. Collagen synthesis abnormality has been also linked to decreased amount of sulfated glycosaminoglycan, especially heparan sulfate, on the stromal cell surface [156–158]. Although many studies (Table 1) have demonstrated contradictory expression levels of the ECM components, these studies indicate the possible disruption in the molecular mechanisms regulating ECM homeostasis.

### 3.4. Degradative Enzymes and Their Inhibitors

A balanced equilibrium between degradative enzymes and their inhibitors is required for microenvironment homeostasis. Many KC studies have documented that the disruption of this homeostasis is due to an upregulation of the degradative enzymes and a downregulation of their inhibitors. It is thought that this disruption may mediate the pathological progression of KC through degradation of the ECM in the cornea, resulting in corneal thinning [46, 159, 160].

Lysosomal enzymes, such as acid esterases, acid phosphatases, and acid lipases, have been shown to have higher expression in the epithelium, stroma, and endothelium of patients with KC [161]. Moreover, cathepsins B and G, which are proteases in lysosomes and activate caspases, have been shown to have elevated expression within the keratocytes of KC corneas [101, 159]. Cathepsin B has also been shown to be overexpressed in tears of KC patients, and it has been proposed that these cathepsins may play a vital role in apoptosis of keratocytes in KC [162].

Matrix metalloproteinases (MMPs) have also been reported to be highly upregulated in KC (reviewed in [163]). MMPs are a large family of calcium-dependent, zinc-containing endopeptidases. MMPs are classified according to substrate preference into subfamilies including collagenases, gelatinases, stromelysins, matrilysins, and membrane-type MMPs [164]. Under normal physiological conditions, MMPs are minimally expressed and are responsible for tissue remodeling and degradation of the ECM [165]. Many studies have reported altered expression of MMPs in KC (reviewed in [160, 163, 166]). Shetty et al. showed that MMP-9 was significantly overexpressed (along with IL-6 and TNF-*α*) in patients' corneal epithelial cells [167]. Interestingly, this upregulation of MMP-9, IL-6, and TNF-*α* was reversed successfully upon treatment with Cyclosporine A (immunosuppressant drug), which may help in arresting disease progression [167]. MMP-9 has also been shown to be elevated in tears of KC [167, 168]; however, it had normal expression in subclinical KC patients [168]. Another study showed elevated levels of MMP-1, MMP-3, MMP-7, and MMP-13 in KC patients' tears [169]. The proteinase inhibitors, on the other hand, have been reported to be downregulated in KC. These inhibitors mainly include *α*1-protease inhibitor, *α*2-macroglobulin, and tissue inhibitors of MMP [160, 170, 171].

### 3.5. Inflammation and KC

Various factors have been suggested to cause inflammation for KC patients. Contact lenses, used as treatment for mild to moderate KC, have been reported to cause the elevation of proinflammatory cytokines in KC patients' tears [172, 173] and to cause dry eye exacerbation [174]. Abnormal eye rubbing has been hypothesized to increase KC progression via aggravating corneal deformities and inflammation [175–177]. Although CXL is considered as an effective treatment for moderate to severe KC [126, 178], acute inflammatory response, allergic conjunctivitis, and bacterial infection have been reported after CXL [179–181]. Examining tears from patients with KC or those who underwent CXL treatment, Balasubramanian et al. have found significant elevations in many cytokines (IL-4, IL-5, IL-6, IL-8, TNF-*α*, and TNF-*β*) in tears from KC patients and only significant elevation of TNF-*α* in the CXL treated group compared with controls [169]. Recruitment of immunoinflammatory cells (macrophages, leucocytes, and antigen presenting cells) has been observed in the epithelium and stroma of keratoconic cornea [182]. A known marker for systemic inflammation (neutrophil to lymphocyte ratio) has been found to be significantly higher in the serum of patients with progressive KC [183]. Additionally, TGF-*β*2 has been found to be elevated in both aqueous humor and corneal epithelial cells in KC [184, 185]. Basal epithelial cells of keratoconic cornea showed moderate-to-strong immunoreactivity for hepatocyte growth factor and its receptor (c-met) [186]. These data indicate that alterations in homeostasis may be attributed to dysregulation in inflammatory mediators, such as cytokines (IL-6, IL-1, IL-17, and TNF-*α*) and growth factors (TGF-*β*, VEGF, and NGF), supporting the potential involvement of chronic inflammation in the pathogenesis of KC [6, 7].

### 3.6. Oxidative Stress

Cellular stress is induced by a sudden disruption of the cellular physiological local environment, compromising cell survival. Through various mechanisms, cells attempt to remove stressors, decrease damage, and maintain or reestablish homeostasis. However, various internal deleterious changes can happen during this process [187]. In 2003, Kenney and Brown hypothesized the existence of a relationship between corneal defects and the scavenging of reactive oxygen species. This was associated with the progressive events in KC that eventually led to oxidative corneal tissue damage [46]. Many proteins that are involved in free radical detoxification, such as glutathione, paraoxonase 1, catalase, superoxide dismutase, and superoxide glutathione, have been shown to have decreased activity in KC [188, 189].

#### 3.6.1. Aldehyde Dehydrogenase

One of the important detoxifying enzymes that may be involved in KC is aldehyde dehydrogenase 3 (ALDH3). ALDH3 is dimeric zinc metalloenzyme that catalyzes the reversible oxidation of alcohols to aldehydes. ALDH3 accounts for approximately 20–40% of the soluble protein content in corneal epithelial cells of mammals (reviewed in [190]). ALDH3 directly absorbs UV light and removes cytotoxic aldehydes produced by UV-induced lipid peroxidation [191]. Mice with defective ALDH3 have been reported to be susceptible to UV-induced corneal clouding [192]. Lack of ALDH3 may lead to lipid peroxidation through UV-induced oxidative destruction of cell membranes and accumulation of cytotoxic aldehydes, such as malondialdehyde (MDA) [193]. A significant and distinct staining for MDA has been identified in 26 corneal tissues with KC but not in healthy tissues [194]. This data suggests that the presence of MDA in the KC corneal tissues may result from low expression of ALDH3 in these samples [194]. Another ALDH member, ALDH1B, has been reported by Mootha et al. to have a 212-fold reduced expression levels of both mRNA and protein in fibroblasts from KC patients [195].

#### 3.6.2. Oxidoreductase

Oxidoreductases catalyze the transfer of electrons from electron donors to electron acceptors, and many of them have been identified as potential sources of superoxide anions in mammalian cells [187]. NADPH dehydrogenase is an oxidoreductase catalyzing the production of reactive oxygen species (ROS) [196]. Studies have discovered that acute exposure of keratinocytes to UV can lead to rapid activation of NADPH dehydrogenase and generation of ROS, which may have distinct physiologic importance [197–199]. NADPH dehydrogenase may represent a cellular “alarm system” that can alert and prime the cells to either adapt to the stress or undergo apoptosis [187]. It has been reported that there is a 7-fold decrease in the expression of NADPH dehydrogenase and NADPH menadione oxidoreductase in KC-affected corneal epitheliums [155]. This significant expression reduction may be one of the pathways through which UV rays affect the progression of KC.

#### 3.6.3. Mitochondrial DNA

Another consequence of corneal oxidative stress is damage to mitochondrial DNA (mtDNA), which has been previously observed in KC corneal tissue [200]. mtDNA is a covalently closed, double-stranded molecule and is located in close proximity to the respiratory chains, which are the main cellular source of ROS. Mitochondrial dysfunction and mtDNA damage, in response to oxidative stressors, have been identified in cultured KC fibroblasts [201]. In human colorectal carcinoma cells, oxidative stress has been reported to lead to the degradation of mtDNA [202].

### 3.7. Lactoferrin/Transferrin

Fleischer's ring, or iron deposition at the base of the cone in the cornea, is a common clinical sign of KC [203]. Physiologically, healthy corneas need iron for the completion of the citric acid cycle and production of ATP, and iron is also an essential component of the rate-limiting enzyme in DNA synthesis [204]. Because iron is necessary in many corneal functions, disruptions in iron homeostasis and elevations in its level can lead to corneal disease [205]. Iron is present extracellularly in the tear film on the surface of the cornea. Iron is carried by two iron binding glycoproteins, lactoferrin and transferrin. These glycoproteins are found in many mucosal fluids, including tears [206, 207]. Through binding to iron, lactoferrin helps regulate iron levels, prevent oxidative damage, and strengthen the cornea's antibacterial defenses [205]. Decreased expression of lactoferrin has been reported in KC corneal epithelial cells [152]. Several studies have also reported lower expression of lactoferrin in the tears collected from KC patients [169, 208, 209], and transferrin has been shown to be expressed at lower levels in the corneal stroma in patients with KC [155]. All these data indicate that reduced expression of iron binding proteins may contribute to deposition of iron in the cornea of KC patients.

Although numerous studies have been done to either investigate the mechanism underlying the progression of KC or report the histopathological changes in KC corneas, some of the results are contradictory. This could be due to many factors. The first factor is inconsistency in the different types of cornea samples, such as whole cornea, epithelium, stromal layer, or keratocytes, used in the various studies. The second factor is the usage of original host tissues versus cultured cells from KC cornea, and the third factor is the variation in the experimental platforms used to investigate the expression of selected proteins. For example, quantifying a specific protein known to be expressed among the different corneal layers could be misleading, because the expression of specific proteins in the different corneal layers could vary during the progression of KC. To better understand KC, we need to standardize the reporting of expression levels for our target and their location within the cornea, especially since previous studies have identified abnormal location and expression of certain proteins in different corneal layers in KC (Table 1). This underlines the importance of taking into account the part of the cornea used for the analysis as the differential protein expression differs from one layer to another.

## 4. Hormones in KC

Sex steroid hormones, namely estrogen, progesterone, and testosterone, are produced by ovaries in females and testes in males. Although they circulate through blood, their effects rely on the receptors present in specific tissues and organs. These receptors are widely expressed in different ocular tissues, including the cornea. Corneal tissues express estrogen receptors types *α* and *β*, progesterone receptors, and androgen receptors. However, the mechanism through which hormones regulate corneal homeostasis still remains unclear (reviewed in [210]).

Previously, the development of KC has been proposed to be correlated with the hormonal changes that occur during puberty, pregnancy, or menopause [211–213]. However, the clinical information related to sex hormones for patients with KC is often limited, presenting a significant barrier to further study. In 2010, Fink et al. studied the effects of gender and hormone status on the severity and progression of KC in both men and women over a 3-year period [214]. This study grouped women into hormone-active and hormone-inactive groups during menopausal transition but failed to identify any significant difference in KC progression between these groups [214].

Conversely, there have been other reported cases in which pregnancy has induced the progression of KC [212, 213, 215], and some studies have postulated that pregnancy may be considered a risk factor for KC [212, 215]. The hormonal changes that occur during pregnancy had a negative impact on corneal biomechanics, as measured by changes in corneal topography [212, 215]. Hoogewoud et al. have reported that, during the gestational period, women have experienced a significant progression in KC, as indicated by a decrease in corrected distance visual acuity (CDVA) and reversible fluctuations in corneal topography [213]. Another large study has reported a similar decrease in CDVA during pregnancy, but changes returned to normal postpartum once the lactation period ended [216]. Women's use of contraceptives has also been reported to have an effect on the curvature of the cornea [217, 218].

Based on the expression pattern of *α* and *β* estrogen receptors in corneal cells, it has been postulated that estrogen is supplied through tears and aqueous humor at concentrations that are approximately half the concentrations found in plasma [219]. The proposed mode of action of these steroidal hormones is via the regulation of gene expression in the nucleus, leading to changes in the concentration of ECM proteins, which are critical to the maintenance of corneal integrity [220]. It is plausible that estrogen may be responsible for weakening the cornea via the stimulation of MMPs and the release of prostaglandins, causing activation of proteolytic enzymes for collagen, disruption of collagen networks, and reduction in corneal-stiffness [220]. Recently, a study reported progression of KC in 6 eyes of 3 women after receiving an in vitro fertilization treatment which increases their estrogen levels [221]. Similarly, a recent study has identified a significant elevation in salivary dehydroepiandrosterone sulfate (DHEA-S, a common precursor to other androgens) levels and a decrease in estrone (a natural estrogen) level in KC patients independent of gender [222]. Elevated DHEA-S possibly increases the expression of specific cytokines (IL-16 and stem cell factors) by blocking endogenous glucocorticoid activity and stimulating the progression of KC [222]. However, no correlation has been detected between the increased salivary DHEA-S level and increased severity of KC [222].

Progesterone hormone, on the other hand, inhibits the prostaglandins that stimulate collagenases. Therefore, it is plausible that the stabilization in the cornea biomechanics during the last half of a normal pregnancy may be due to the action of progesterone, suggesting that progesterone may have a protective effect against the progression of KC during pregnancy [223]. Since there is a correlation between changes in corneal physiology and elevated levels of estrogen, KC may be triggered by elevated levels of estrogen coupled with a genetic disposition to a weaker cornea.

Moreover, some studies correlated KC progression with thyroxine hormone [224–227]. It was found that thyroxine levels were higher in tears of KC patients independent of their serum thyroxine level [225, 227]. Thyroxine has important roles in the differentiation, growth, metabolism, and physiological function of almost all tissues, including the cornea [228–230].

## 5. Summary 

**Figure 4:**
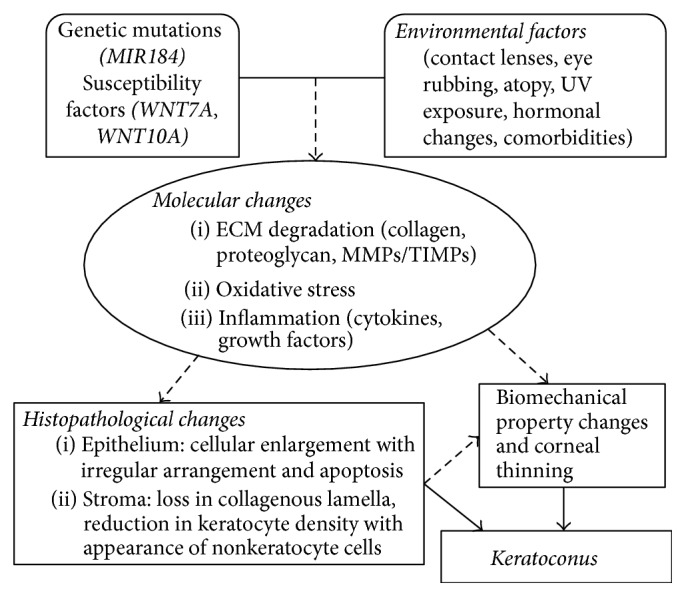
A potential physiological model for the pathogenesis of keratoconus.

Keratoconus is a complex disorder with both genetic and environmental factors and may present as a secondary phenotype associated with other disorders. The disease progression of KC affects the epithelium, Bowman's layer, stroma, and Descemet's membrane of the cornea, but not the corneal endothelium. Extensive research in the histopathology of KC has provided critical information about the cellular and molecular mechanisms of KC pathogenesis (Figure 4). A number of proteins in several different pathways have been identified to have altered protein abundance in KC-affected cornea samples (Figure 4). The primary corneal layer(s) with these abundance alterations will need to be determined. The lack of replication between different studies might be due to the following variables: different stages of KC, sample size, detection technique, and statistical tools for data analysis. Animal models of KC, which are currently lacking, will significantly promote our understanding of the pathogenesis of KC. A better understanding of the proteins and pathways involved in KC histopathogenesis may provide potential therapeutic targets for disease prevention and early diagnosis, thus delaying or arresting its progression and improving treatment of this severe vision threatening disorder.

## Figures and Tables

**Table 1:** List of proteins with expression change in cornea samples affected with keratoconus.

Protein name	Functions	Corneal layer	Method of detection	Expression change	Reference
Superoxide dismutase	Antioxidant enzyme that can metabolize superoxide radicle	Central portion of the cornea	ELISA	Decreased	[188]
Annexin A2	Involved in cellular growth regulation and in signal transduction pathways	Epithelium	2D-DIGE	Decreased	[155]
Annexin A8				Increased	
Carbonic anhydrase I	Playing a role in the barrier function of corneal endothelium	Stroma	Nano-ESI-LC-MS (MS)^2^	Decreased	[152]
Collagen I *α*1, Collagen I *α*2	Structural protein	Epithelium	Nano-ESI-LC-MS (MS)^2^	Decreased	[152]
Cathepsin B	Member of corneal epithelial lysosomal proteases	Epithelium and stroma; tears	IM; MF10-LTQ-FT MS	Increased	[159, 162]
Vimentin	A type of intermediate filament	Stroma; epithelium	Nano-ESI-LC-MS (MS)^2^	Increased	[152, 155]
Keratocan	Proteoglycan protein, unique for cornea	Stroma	IM, Nano-ESI-LC-MS (MS)^2^	Increased	[154, 155]
Serotransferrin	Iron binding transport proteins	Stroma	Nano-ESI-LC-MS (MS)^2^	Decreased	[155]
Transketolase	Enzyme in the nonoxidative branch of the pentose-phosphate pathway	Epithelium	Nano-ESI-LC-MS (MS)^2^	Decreased	[155]
Phosphoglycerate kinase 1	ATP-generating glycolytic enzyme	Epithelium	Nano-ESI-LC-MS (MS)^2^	Decreased	[155]
NADPH oxidase	Alarm system for cellular stress response	Epithelium	Nano-ESI-LC-MS (MS)^2^	Decreased	[155]
NADPH menadione oxidoreductase 1	Reducing menadione into a stable hydroquinone that can be readily conjugated and excreted	Epithelium	Nano-ESI-LC-MS (MS)^2^	Decreased	[155]
Heat shock B1	Involved in stress resistance and actin organization	Epithelium	2D-DIGE	Decreased	[155]
S100-A4	Binding to several components of the cytoskeleton	Epithelium	WB and IM; 2D-DIGE; Nano-ESI-LC-MS (MS)^2^	Increased	[145, 152, 155]
Keratin 1	Structural protein	Epithelium	Nano-ESI-LC-MS (MS)^2^	Increased	[155]
Keratin 6A	Structural protein	Epithelium	Nano-ESI-LC-MS (MS)^2^	Increased	[152]
Keratin 16	Structural protein	Epithelium	Nano-ESI-LC-MS (MS)^2^	Increased	[152]
Desmoglein 3	Cell adhesion molecule	Epithelium	WB and IM	Increased	[145]
Decorin	Proteoglycan core protein	Stroma	IM; Nano-ESI-LC-MS (MS)^2^	Increased	[154, 155]
Collagen VI *α*1, Collagen VI *α*2, and Collagen VI *α*3	Structural protein	Epithelium	Nano-ESI-LC-MS (MS)^2^	Decreased	[152]
Collagen VII *α*1	Structural protein	Epithelium	Nano-ESI-LC-MS (MS)^2^	Decreased	[152]
Lactoferrin	Iron binding transport proteins	Tears; epithelium	2D-DIGE; Nano-ESI-LC-MS (MS)^2^	Decreased	[152, 208, 209]
Lipocalin 1	Major lipid-binding protein in tears	Stroma	Nano-ESI-LC-MS (MS)^2^	Decreased	[152]
Hepatocyte growth factor	Regulating cell growth and motility	Epithelium	IM	Increased	[186]

Nano-ESI-LC-MS (MS)^2^: Nano-Electrospray Ionization Liquid Chromatography Tandem Mass Spectrometry; 2D-DIGE: two-dimensional-difference gel electrophoresis coupled with mass spectrometric methods; IM: immunostaining; WB: Western blot; MF10-LTQ-FT MS: prefractionating and enriching the proteins followed by linear ion trap quadrupole Fourier transform mass spectrometer.
